# The Chemical Composition and Baking Quality of Rye Flour from Grain with Organic Production

**DOI:** 10.3390/foods15010003

**Published:** 2025-12-19

**Authors:** Sylwia Stępniewska, Grażyna Cacak-Pietrzak, Anna Fraś, Magdalena Wiśniewska, Katarzyna Sujka, Justyna Grabarczyk, Dariusz Dziki

**Affiliations:** 1 Department of Food Technology and Assessment, Division of Fruit, Vegetable and Cereal Technology, Institute of Food Sciences, Warsaw University of Life Sciences (WULS—SGGW), Nowoursynowska 159C Street, 02-787 Warsaw, Poland; grazyna_cacak_pietrzak@sggw.edu.pl (G.C.-P.); katarzyna_sujka@sggw.edu.pl (K.S.); 2 Department of Grain Processing and Bakery, Prof. Wacław Dąbrowski Institute of Agricultural and Food Biotechnology—State Research Institute, Rakowiecka 36 Street, 02-532 Warsaw, Poland; justyna.grabarczyk@ibprs.pl; 3 Plant Breeding and Acclimatization Institute—National Research Institute, Radzików, 05-870 Błonie, Poland; a.fras@ihar.edu.pl (A.F.); m.wisniewska@ihar.edu.pl (M.W.); 4 Department of Thermal Technology, Lublin University of Life Sciences, Głęboka 31 Street, 20-612 Lublin, Poland

**Keywords:** falling number, non-starch polysaccharides, rye flour, water absorption, dietary fiber

## Abstract

The aim of this study was to evaluate the influence of rye genotype, harvest year, and growing location on the chemical composition and baking quality of rye flour (55% extraction rate). Flours were produced from five population cultivars and two hybrid cultivars, cultivated in two locations in Poland, Osiny and Grabów, during the 2018/2019 and 2019/2020 seasons under organic farming conditions. Basic chemical composition (lipids, minerals, protein, carbohydrates), dietary fiber and its fractions (non-starch polysaccharides and lignin), and water extract viscosity were determined. Baking quality was assessed using falling number and water absorption. The results showed that harvest year exerted the strongest effect on rye flour properties. Flour produced from grain harvested in 2019 contained higher levels of protein (7.9% d.m.), lipids (0.74% d.m.), nutrition compounds, and falling number (297 s) but lower water absorption (63.3%). Rye flour samples from the 2020 harvest had a higher proportion of soluble fractions, which increased water extract viscosity. Among the cultivars, Dańkowskie Skand demonstrated the most favorable baking characteristics, with the lowest falling number (271 s) and the highest water absorption (65.5%). The most advantageous chemical components were observed in Dańkowskie Hadron flour due to its high contents of dietary fiber (7.47% d.m.), non-starch polysaccharides (6.63% d.m.), lignin (0.83% d.m.), and elevated water extract viscosity (5.21 mPa·s). Hybrid cultivars were characterized by lower protein content and lower amylolytic activity, while no significant differences between hybrid and population cultivars were found in terms of dietary fiber and its components.

## 1. Introduction

Common rye (*Secale cereale* L.) is one of the main cereals grown in the temperate zone, primarily in central and north-eastern Europe [1,2]. According to USDA data [3], global rye production in the 2024/2025 season was estimated at 11.1 million tons, of which 7.4 million tons (66% of total production) was harvested in EU countries. The group of the world’s largest rye producers includes Germany (3.1 million tons), followed by Poland (2.3 million tons) and Russia (1.2 million tons).

Currently, one of the priorities of the European Union’s agricultural policy (European Green Deal) is to reduce the harmful impact of agriculture on the natural environment by promoting the greater use of natural and ecological methods of crop production. One of the main goals of this strategy is to increase the area of organic farming to 25% of the total agricultural land in the EU by 2030 [4]. Rye is considered one of the most suitable cereals for cultivation under organic farming systems, where the use of mineral fertilizers and chemical plant protection products is prohibited. The cultivation requirements of rye are relatively low compared with other winter cereals due to its high tolerance to nutrient-poor soils and abiotic stresses. Rye can be cultivated on light soils that are low in nutrients (N, P, K) and acidic [5]. Its well-developed root system enables efficient uptake of nutrients from deeper soil layers, leading to reduced mineral fertilization needs. Rye is highly resistant to low temperatures and drought, which stabilizes yields without the use of synthetic growth regulators or mineral fertilizers [6]. Rye germinates quickly and tillers intensively, which effectively limits weed infestation in the crop. Furthermore, rye exhibits naturally high resistance to many fungal diseases, reducing the need for chemical protection [2]. In organic crop rotations, rye additionally serves as a soil-improving crop that enhances the phytosanitary status of the field and performs well as a preceding crop for spring cereals and legumes [7].

One of the primary applications of rye kernel is processing into refined and wholemeal flours, which are the primary raw materials in the baking industry for producing rye and mixed breads (rye–wheat, wheat–rye). They are also used in the confectionery industry as an additive to wheat flour in the production of gingerbread and crackers [8].

Rye flour’s chemical composition varies according to the grain and the milling system (simple or complex). The composition of wholemeal flour obtained through simple milling corresponds to that of the entire grain [8,9]. Quantitatively, its main components are saccharides, including primarily starch and non-starch polysaccharides (NSPs), followed by proteins, lipids, minerals (ash), and vitamins of the B complex [10]. In refined flours, the removal of the outer kernel layers during milling alters the proportion of individual components, which affects the nutritional value of the final products. As progressively larger portions of the outer layers are removed, the flour contains decreasing amounts of protein, minerals, and non-starch polysaccharides, while its starch content increases [9].

It should be emphasized that rye products, particularly bread, are an abundant source of bioactive constituents, particularly dietary fiber. The major constituents of rye fiber are non-starch polysaccharides (NSPs), such as arabinoxylans, fructans, β-glucans, and cellulose. Fiber also contains lignin and NSP-associated waxes, saponins, tannins, phytates, and phenolic compounds, including phenolic acids and alkylresorcinols [11,12]. Dietary fiber plays a significant role in the human diet, both through its beneficial effects on the digestive system and its preventive role in reducing the risk of chronic non-communicable diseases [13].

The baking suitability of rye flour is largely defined by its main components, i.e., starch and non-starch polysaccharides, as well as the enzymes involved in their decomposition. The key technological indicators for assessing the baking value of rye flour include the falling number and water absorption, as they directly reflect the flour’s ability to form dough with the appropriate consistency and its susceptibility to amylolytic degradation [14]. Rye flours used in baking must exhibit appropriate baking quality, including flours produced from organically grown grain. Considering that plant breeding stations do not develop rye varieties specifically dedicated to organic cultivation, farmers practicing organic rye production use seeds of varieties adapted to intensive agricultural technology. This leads to difficulties in obtaining grain and, consequently, flour of a quality suitable for processing.

To the best of our knowledge, studies addressing the influence of environmental and genetic factors on the chemical composition and baking quality of flours obtained from organically grown rye are scarce, which underscores the need for conducting such research. Therefore, the was aim of this study was to evaluate the effects of environmental and genotype factors on the chemical composition and selected baking indicators of low-extract flour from the most commonly grown rye varieties in Poland, cultivated on organic farms. It was assumed that the conducted research, in addition to expanding scientific knowledge, would also have a practical aspect and allow for the selection of rye varieties best suited to organic production conditions.

## 2. Materials and Methods

### 2.1. Materials

The tested material consisted of rye flour with a low extraction rate, obtained through laboratory milling of grains from two hybrid and five population rye cultivars cultivated under organic production conditions. The rye grains were collected from two sites in Poland, Grabów and Osiny, across the 2018/2019 and 2019/2020 growing seasons. Site characteristics, agronomic practices, and meteorological conditions during rye cultivation are fully detailed in the work of Stępniewska et al. [15].

### 2.2. Methods

#### 2.2.1. Milling Procedure

Prior to milling, rye grain was cleaned using a Granotest device (Brabender, Germany) and conditioned to a moisture content of 14.0%. Milling was performed using a two-pass laboratory mill the Quadrumat Senior (Brabender, Germany), with a 2.0 kg grain sample. During milling, the room was maintained under the following environmental conditions: a temperature of 20 ± 2 °C and a relative humidity of 60–70%. The milling process produced four fractions: breaking flour, reduction flour, breaking bran, and reduction bran. Each of the obtained fractions was weighed separately using a technical balance, and the yield was calculated relative to the grain mass. To standardize the conditions for further analyses, breaking flour was added to the reduction flour in an amount sufficient to achieve a composite flour yield of 55%. The Supplementary Section included the results of milling (Table S1), taking into account the yields of the obtained flour and bran, as well as the composition of the flour used as material for study.

#### 2.2.2. Major Chemical Constituents

The major chemical composition of the analyzed rye flour samples was determined based on basic components such as moisture, ash, fat, protein, and carbohydrate contents, as well as dietary fiber content and its components.

Moisture content was determined following ISO 712 [16], ash content according to ISO 2171 [17], fat content according to ISO 11085 [18], and protein content according to ISO 20483 [19]. The carbohydrate content was calculated as the difference between 100% and the sum of the other constituents. All analyses were performed using the equipment described by Stępniewska et al. [15].

Dietary fiber (DF) content was determined by an enzymatic–chemical method according to AACC 32–25 [20] and AOAC 994.13 [21] standards, calculated as the sum of non-starch polysaccharides (NSPs) and lignin with associated polyphenols. NSP content, was determined as a soluble fraction (S-NSP) and an insoluble fraction (I-NSP) by gas chromatography (GC) according to the method of Englyst and Cummings [22]. Both non-starch polysaccharide fractions were calculated as the sum of individual monomers: arabinose, xylose, mannose, galactose, and glucose. In the first step, the analyzed research samples were subjected to enzymatic hydrolysis of starch, then centrifuged and separated into soluble and insoluble fractions. Each of the obtained polysaccharide fractions was hydrolyzed with 1 M sulfuric acid (100 °C, 2 h) to monosaccharides, which were then converted to volatile alditol acetates. The alditol acetates were separated on an Rtx-225 capillary column (0.53 mm × 30 m) using a Clarus 600 gas chromatograph (PerkinElmer, Waltham, MA, USA) equipped with an autosampler, a splitter injection port, and a flame ionization detector. Assay conditions: helium carrier gas, flow rate of 2 mL/min, injector and detector temperature of 275 °C. Column temperature program: initial temperature of 185 °C, 1 min; increase 5 °C/min to 215 °C; isotherm 215 °C, 10 min.

Lignin and other insoluble residues were determined gravimetrically according to Theander and Westerlund [23]. According to the method, the sample was digested with 72% sulfuric acid, and after drying (105 °C, 16 h), the residue was combusted (550 °C, 5 h). The percentage of lignin was calculated based on the mass loss after combustion of the dried insoluble material.

#### 2.2.3. Viscosity of Water Extract

The viscosity of aqueous extracts of rye flour (WEV) was determined according to the methodology of Boros et al. [24]. The suspension of aqueous extracts was incubated for 1 hour at 30 °C and then centrifuged. The viscosity of the obtained supernatant was measured using a Brookfield LVDV-II+ cone/plate viscometer (Brookfield, Stoughton, MA, USA) equipped with a cone spindle an angle of 0.8°. The measurement was performed at a constant temperature of 30 °C. WEV results are expressed in mPa·s.

#### 2.2.4. Technological Parameters

##### Falling Number

The falling number (FN) was determined according to ISO 3093 [25] using the equipment described by Stępniewska et al. [26].

##### Water Absorption

The water absorption (WA) of the tested rye flour samples was measured using a Mixolab (Chopin Technologies, Villeneuve-la-Garenne, France) according to following ISO 17718 [27].

#### 2.2.5. Statistical Analysis

Statistical analysis was carried out using the Statistica 13.3 PL software (StatSoft, Cracow, Poland). All measurements were conducted in at least three repetitions. To evaluate the effect of factors related to the origin of the flour, including harvest year, rye cultivar, and growing location, on the properties of the tested flour samples, a three-way ANOVA was applied. Differences among the mean values were verified using Tukey’s post hoc test. The level of significance was set at α = 0.05 and α = 0.01. To assess the extent of variation among flours and identify the factor with the strongest impact, principal component analysis (PCA) was employed. This analysis was based on mean values for each flour, which corresponded well with the results obtained for individual replicates.

## 3. Results and Discussion

### 3.1. Major Chemical Constituents of the Flour Samples

#### 3.1.1. Moisture, Protein, Ash, Fat, and Carbohydrate Content

Flour moisture is a key quality parameter affecting the storage stability of rye flour. Elevated moisture levels create favorable conditions for the development of bacteria and fungi, which in turn increases the risk of mycotoxin production and deterioration of flour quality [28]. Flours intended for safe long-term storage should maintain moisture levels well below about 14.0% to inhibit mold growth. Lower moisture levels reduce water activity, inhibit microbial growth, and thereby contribute to prolonging shelf life and maintaining the product’s technological functionality [29]. In the current study, the moisture content ranged from 12.4% to 14.9% (Table 1). Although all rye grain samples were conditioned to the same level of moisture and milled under identical laboratory conditions, the resulting flours exhibited significant differences in moisture content. Most samples of tested rye flour exhibited moisture content below 14.0%, while one sample reached 14.4% (rye flour from grain cultivar Dańkowskie Hadron). It was observed that, despite applying identical tempering conditions, the extent of water penetration and redistribution within rye kernels may differ. This variability is likely associated with characteristics of the grain, such as differences in kernel structure, degree of vitreousness, thickness of bran layer, and water-binding capacity, which influence the way moisture is absorbed and retained during the conditioning process [30]. The current study demonstrated a clear impact of all determined factors on the discussed quality attributes. Rye flour produced from rye grain harvested in 2019 was characterized by significantly higher moisture content (average 14.3%) compared to flour from rye grain harvested in 2020, for which the average moisture content was significantly lower, i.e., 13.5%. The study showed that flour produced from rye grain from the Osiny location was characterized by an average moisture content 0.2 percentage points higher than flour from rye grain from the Grabów location (average 13.8%). It was also found that flour made from the rye grain Dańkowskie Skand population cultivar had significantly the lowest moisture content (average 13.6%), while flour made from rye grain of the population cultivar Dańkowskie Turkus had the highest moisture content (average 14.4%).

According to previous studies, the protein content in rye flour is lower than that in wheat flour; however, rye proteins show a higher biological value, which has been attributed to a higher share of soluble protein fractions [31,32]. By the end of the acid fermentation of rye dough, most of the proteins become water-soluble. This process affects dough yield and its degree of acidification, as well as crumb elasticity, bread volume, and taste [33]. In the analyzed rye flour samples, the protein content ranged from 5.3 to 6.1% d.m. In previous studies [26], wholemeal rye flours produced in 10 industrial mills across Poland exhibited significantly higher protein content, ranging from 9.2 to 14.0% d.m. This is due to the presence of all anatomical parts of the grain in wholemeal flours, whereas the flours analyzed in the present study were largely deprived of particles from the outer grain layers, which are rich in this nutrient [9]. All three experimental factors had a significant effect on the protein content. Flour from rye grain harvested in 2019 had significantly higher protein content (average 7.9% d.m.) than flour from grain harvested in the following year (average 6.2% d.m.), which corresponds with earlier observations regarding the composition of the raw material used for rye flour production [15]. Significantly higher protein content was also observed in flour obtained from rye grain grown in the Grabów location (average 7.4% d.m.) compared to the Osiny location (average 6.8% d.m.). Moreover, flours from hybrid rye cultivars showed significantly lower protein content (range 6.6–6.8% d.m.), whereas flours from population rye cultivars exhibited higher levels (range 7.1–7.3% d.m.). The literature repeatedly shows that hybrid rye cultivars, although generally characterized by higher yield potential, usually have lower protein content compared to population cultivars [15,34,35,36]. According to Oest et al. [37], during rye bread production, proteins can form complexes with hemicelluloses, which limit protein denaturation and water release, negatively affecting starch gelatinization and the final quality of rye bread. Therefore, the lower protein content in flours from hybrid rye cultivars may reduce the formation of protein–hemicellulose complexes, potentially improving starch gelatinization; however, the limited protein content, by reducing the dough’s water-binding capacity, may negatively affect crumb elasticity [38]. These interactions suggest that the choice of rye cultivar can have a dual effect on dough behavior and bread quality, where higher starch gelatinization may be accompanied by lower structural stability of the crumb. Further studies are needed to quantify the balance between protein content, water-binding capacity, and starch gelatinization in different rye cultivars, which could provide practical guidance for optimizing flour selection in specific baking applications.

The ash content in rye flour may reflect the level of minerals of significant metabolic importance. However, their bioavailability depends on the presence of accompanying substances, such as phytates and dietary fiber, which can form complexes with calcium, iron, or zinc ions, thereby limiting their absorption [39]. The ash content in the examined flour samples fell between 0.55 and 1.01% d.m. (Table 1). Higher values (ranging from 0.75 to 1.52% d.m.) were previously reported for high-extract rye flours obtained from milling companies located in Poland [14]. Statistical analysis showed that all experimental factors had a significant effect on this component. The most pronounced influence was observed for the harvest year—flours produced from rye grain collected in 2020 exhibited notably higher ash content (mean 0.85% d.m.) compared to rye flour samples obtained from the 2019 harvest year (mean 0.62% d.m.). The effect of harvest year may result from differences in weather conditions, including rainfall and temperature during grain ripening. Moreover, rye flours obtained from grain growing in Grabów exhibited higher ash content (mean 0.78% d.m.) than those from Osiny (mean 0.70% d.m.). These differences could be attributed to variations in soil conditions and agronomic practices between cultivation sites [15]. Flour samples produced from hybrid rye cultivars were characterized by significantly higher ash content (the mean values for the cultivars Tur and KWS Dolaro were 0.77% and 0.76% d.m., respectively), whereas flour obtained from population rye cultivars showed lower ash content, ranging from 0.71% d.m. for the cultivars Dańkowskie Granat and Dańkowskie Turkus to 0.75% d.m. for Dańkowskie Hadron. These observed differences in ash content may result from a combination of factors, including genetic variation between cultivars, environmental conditions during grain development (such as soil mineral composition, rainfall, and temperature), and agronomic practices at different growing locations. Additionally, differences in grain structure, kernel size, and the proportion of outer layers retained during milling may also contribute to variations in mineral content, as ash is predominantly concentrated in the bran and aleurone layers of the grain.

The fat content in flour showed values ranging from 0.59 to 0.85% d.m. (Table 1). Higher fat content was observed in low-extract and wholemeal rye flour samples studied by Bucsella et al. [40] (means of 1.15 and 3.07% d.m., respectively). It should be noted that, although the fat content in rye flours is generally low, it is characterized by a favorable nutritional profile due to the elevated proportion of unsaturated fatty acids, including linoleic, oleic, and linolenic acids [41]. In the present study, all experimental factors had a significant influence on the fat content, with the cultivar factor showing the greatest effect. Similar to protein content, flours obtained from hybrid rye cultivars had significantly lower fat content (the average values for the Tur and KWS Dolaro cultivars were 0.70 and 0.65% d.m., respectively). Flours from population cultivars exhibited significantly higher fat content (except Dańkowskie Turkus), possibly due to a higher proportion of germ and aleurone layers in the grain, which are naturally rich in lipids. From a practical perspective, the higher fat content in flours from population cultivars may influence not only their nutritional value but also the technological properties of the dough, such as elasticity and moisture retention capacity. Conversely, flours from hybrid cultivars, characterized by lower fat content, may be more advantageous in products where extended shelf life and reduced susceptibility to lipid rancidity are desired. These findings underscore the importance of selecting the appropriate grain cultivar according to the intended application of the flour and indicate that differences in chemical composition between cultivars can have a measurable impact on the quality and properties of the final product.

Carbohydrate content in the analyzed rye flours ranged from 82.4 to 86.2% d.m. (Table 1), indicating that it is the main component of these samples. These values were significantly higher than the literature data for wholemeal rye flour, where the carbohydrate content typically ranges from 56 to 70% d.m. [42]. These differences may result from the degree of milling: in the present study, flours with a higher proportion of endosperm were analyzed, whereas wholemeal flours contain a substantial portion of the fruit coat, which is rich in fiber and minerals but low in starch [43]. The statistical analysis indicated that all experimental factors had a significant effect on this nutrient. Significantly higher carbohydrate content was observed in rye flour samples obtained from grain harvested in 2020 (average 84.7% d.m.) and in flours from rye grown in the Osiny location (average 84.9% d.m.). Furthermore, flour from the hybrid rye cultivar KWS Dolaro had the highest carbohydrate content (average 84.9%), whereas flours from population cultivars exhibited lower carbohydrate content, ranging from 83.7 to 84.3% d.m.

#### 3.1.2. Dietary Fiber Content and Its Components

Results concerning the dietary fiber content and its components for the analyzed rye flour samples are shown in Table 2. Dietary fiber (DF) consists of carbohydrate polymers containing at least three monomeric units that are not digested or absorbed in the small intestine [44]. The presence of dietary fiber in the human diet provides numerous health benefits associated with disease prevention. Its beneficial effects are primarily linked to the regulation of lipid and carbohydrate metabolism, as well as gastrointestinal function. As a result, a diet rich in fiber helps reduce the risk of developing conditions such as atherosclerosis, coronary heart disease, type 2 diabetes, and obesity. Moreover, the regular consumption of fiber-rich foods contributes to a reduced risk of colorectal cancer [45,46]. The technological process of rye bread production affects the solubility and content of dietary fiber (DF) components [44]. Szentmiklóssy et al. [47] demonstrated that during breadmaking, total dietary fiber content undergoes only minor changes. However, significant alterations occur in the composition of individual fiber fractions, particularly a marked decrease in the water-extractable fraction, likely due to structural modifications occurring during fermentation and baking. In the analyzed rye flour samples, DF content ranged from 6.24 to 8.63% on a dry matter basis, which was significantly lower than that in the corresponding rye grain [15]. All experimental factors exerted a significant effect on DF content. Flours produced from rye grain harvested in 2019 and from the Grabów location exhibited significantly higher DF content (averages of 7.52 and 7.58% d.m., respectively) compared with flours obtained from grain harvested in 2020 and from the Osiny location (averages of 6.91 and 6.86% d.m., respectively). Considering the rye cultivar, the average DF content in flour from each analyzed cultivar exceeded 7.00% d.m. For comparison, the DF content in rye grain was significantly higher, exceeding 15% d.m. [15]. The lower DF content in flour relative to grain indicates that the milling process leads to partial loss of this component, which is predominantly localized in the outer layers of the grain [9]. No significant differences were found between flours derived from population and hybrid rye cultivars. In flours from population cultivars, DF content ranged from 7.08 to 7.47% d.m., whereas in flours from hybrid cultivars, it ranged from 7.03 to 7.39% d.m. These results suggest that, although milling reduces the overall dietary fiber (DF) content, the relative similarity between cultivars indicates that genetic factors may play a limited role in determining fiber levels in flour. Variations observed between harvest years and locations are likely related to environmental conditions, such as climate and soil properties, which influence the development and composition of the grain’s outer layers. From a practical standpoint, preserving higher fiber content is desirable due to its beneficial effects on human health and its impact on dough viscosity, water retention, and bread texture. Future research could focus on optimizing milling processes and selecting appropriate grain types to minimize fiber loss, as well as investigating the interactions between DF, starch, and protein that ultimately affect the technological and nutritional quality of rye flour products [37]. 

The main component of dietary fiber is non-starch polysaccharides (NSPs), which are polysaccharides other than starch that form part of plant cell walls. NSPs are classified into cellulose and non-cellulosic polysaccharides, also referred to as hemicelluloses. In rye grain and its milling products, a significant portion of NSPs consists of arabinoxylans and β-glucans [47,48,49], which strongly influence flour water absorption, dough yield, and bread volume, as well as crumb firmness and moisture [50].

The total NSP content (T-NSP) in rye grain is higher than in the corresponding flour, because NSPs are predominantly concentrated in the peripheral parts of the grain, which are partially removed during milling [51]. In the current study, the content of T-NSP in rye flours ranged from 5.57 to 7.51% d.m. For comparison, Fraś et al. [49] reported T-NSP values in rye grain ranging from 12.7 to 13.0% d.m. Similar to DF content, higher T-NSP values were observed in flours produced from rye grain harvested in 2019 and from the Grabów location (averages of 6.66 and 6.70% d.m., respectively) compared with flours from grain harvested in 2020 and from the Osiny location (averages of 6.20 and 6.16% d.m., respectively). Regarding the rye cultivar, the average T-NSP content in flour from each analyzed cultivar exceeded 6.65% d.m. No significant differences were found between population and hybrid rye cultivars. In flours from population cultivars, T-NSP content ranged from 6.24 to 6.63% d.m., whereas in flours from hybrid cultivars, it ranged from 6.28 to 6.57% d.m. These findings suggest that, as with total dietary fiber, environmental factors such as year of harvest and cultivation site play a greater role than genetic differences in determining T-NSP content. The observed consistency between DF and T-NSP trends indicates that the distribution of soluble and insoluble polysaccharides responds similarly to environmental conditions and grain development. From a practical perspective, higher T-NSP levels may positively influence dough water retention and viscosity, potentially improving bread texture and volume. Future research could explore the interactions between T-NSP, starch, and protein in different rye cultivars, as well as the impact of milling and processing parameters on preserving these valuable components in flour.

NSPs are divided into soluble (S-NSP) and insoluble (I-NSP) fractions based on their solubility in water [52]. In the present study, I-NSP and S-NSP contents in the analyzed rye flour samples were in the range of 2.10 to 4.22% d.m. and 2.89 to 4.40% d.m., respectively. For comparison, Cyran and Cygankiewicz [53] reported that rye flours with extraction rates of 44–68% exhibited a wider range of I-NSP content (1.0–4.8% d.m.), whereas S-NSP content was higher (4.0–5.7% d.m.). Analysis of NSP fractions indicated clear differences between flour samples produced from rye grain harvested in different years. Flours obtained from grain harvested in 2020 contained a higher proportion of S-NSP, while those from the 2019 harvest were characterized by a slightly higher share of I-NSP. This variability suggests that the harvest year is an important factor affecting not only total NSP content but also the distribution between soluble and insoluble fractions in the flour. Furthermore, the effect of the growing location was significant only for I-NSP content. Flours produced from rye grain grown in Grabów contained significantly higher I-NSP (average 3.34% d.m.) than those from Osiny (average 2.80% d.m.). In contrast, S-NSP content was similar in flours from both locations, averaging 3.36% d.m.

In addition to non-starch polysaccharides, dietary fiber also contains lignin, which is mainly present in the outer layers of the grain, in the fruit coat and aleurone layer; thus, its content in flour is closely related to the degree of milling [54]. Lignin constitutes the insoluble fraction of dietary fiber and influences fecal bulk, the binding of toxins, reduction in cholesterol absorption, and inhibition of starch hydrolysis, and may help prevent constipation [55,56]. In the analyzed rye flour samples, lignin content ranged from 0.45 to 1.12% d.m. For comparison, Fraś et al. [49] reported significantly higher lignin content in rye grain from four cultivars harvested in 2017–2019 (range 2.39–2.45% d.m.). In this study, flours produced from grain harvested in 2019 contained significantly more lignin (average 0.87% d.m.) than those from the 2020 harvest (average 0.71% d.m.). The cultivation location of the rye grain also had a significant effect: flours from grain grown in Grabów exhibited higher lignin content than those from Osiny (averages of 0.88 and 0.70% d.m., respectively). Regarding rye cultivars, the highest lignin content was found in flours from the population cultivars Dańkowskie Hadron and Piastowskie (averages of 0.83 and 0.84% d.m., respectively), whereas the lowest content was observed in flour from the population cultivar Dańkowskie Granat (average 0.72% d.m.). These findings indicate that, similarly to other dietary fiber components, lignin content in flour is influenced more by environmental conditions such as harvest year and cultivation site than by genetic factors. The lower lignin content in flour compared with grain shows that the milling process partially removes this component, which is predominantly located in the outer layers of the kernel. From a technological perspective, lignin contributes to dough structure and water-binding properties, while from a nutritional standpoint, it plays a role in promoting gastrointestinal health. Future research could investigate the interactions between lignin, other fiber fractions, and starch, as well as the effects of different milling and processing techniques on the retention of lignin in rye flour.

### 3.2. The Water Extract Viscosity of Tested Low-Extract Rye Flour

The water extract viscosity (WEV) of the tested rye flour ranged from 3.37 to 6.35 mPa·s (Table 2), which was significantly lower than that of the rye grains from which the flours were produced [15]. This difference is attributed to the lower content of soluble dietary fiber in low-extraction flour, particularly water-extractable arabinoxylans, which are the main contributors to WEV [57]. The study demonstrated a significant effect of all experimental factors on WEV. Rye flour samples from grain harvested in 2020 showed higher WEV (mean 5.13 mPa·s) than those from the 2019 harvest (mean 4.40 mPa·s), likely due to the higher content of the soluble fraction of non-starch polysaccharides in the 2020 grain. The effect of the growing location was less pronounced: flours from grain grown at Grabów exhibited slightly higher WEV than those from Osiny (means of 4.80 and 4.74 mPa·s, respectively). Consistent with the findings of Cyran and Dynkowska [53], flours from hybrid rye cultivars showed significantly lower WEV than those from population cultivars. In this study, WEV of flours from population rye cultivars ranged from 4.68 to 5.21 mPa·s, whereas for hybrid cultivars, it did not exceed 4.44 mPa·s. Analysis of the results reveals that WEV is strongly influenced by the content of soluble dietary fiber, with environmental factors such as harvest year exerting a more pronounced effect than cultivation site. Higher WEV in flours from specific years or cultivars may improve dough water retention, gas-holding capacity, and bread volume, highlighting practical implications for selecting rye flours for particular baking applications. Conversely, the lower WEV observed in hybrid cultivars could limit these technological properties but may be beneficial in products where reduced dough viscosity is preferred. Future research could aim to optimize milling and processing parameters to preserve or enhance soluble fiber content and WEV, as well as to explore the interactions between WEV, dietary fiber, and other flour components that ultimately determine the quality of rye bread.

### 3.3. Technological Parameters of Tested Low-Extract Rye Flour

The technological properties of the analyzed rye flour samples were evaluated in terms of the falling number (FN) and water absorption, and the corresponding results are presented in Table 3.

In the analyzed rye flour samples, the FN, an indicator of amylolytic enzyme activity, ranged from 215 to 343 s. The FN values obtained in the present study indicate low amylolytic activity, which may suggest that doughs prepared from these flours would exhibit poor leavening ability, limited aeration, and produce bread with low volume and a firm crumb [26,37]. Similar observations have been reported in earlier studies [26,58], where it was observed that currently produced rye flours exhibit low amylolytic activity, with FN values generally exceeding the optimal range (i.e., above 200 s) [59]. It should be emphasized that, in comparison to wheat flours, rye flours generally show lower falling number values, due to the higher susceptibility of rye grains to germination, which increases enzymatic starch degradation [60]. In the study by Wysocka et al. [61], wheat flours obtained from grains harvested in 2019–2021 exhibited falling number values ranging from 290 to 505 s. Statistical analysis demonstrated that all experimental factors had a significant effect on FN values. The most pronounced influence was observed for the grain harvest year. Flour samples produced from rye grain harvested in 2019 were characterized by significantly higher FN values (mean of 297 s) compared with flours obtained from grain collected during 2020 harvest (mean of 264 s). Furthermore, flours produced from rye grain originating from Osiny exhibited significantly lower FN values (mean of 274 s) than those from Grabów (mean of 290 s). Flour produced from hybrid rye cultivars (KWS Dolaro and Tur) showed significantly higher FN values (mean of 294 s for both cultivars). In contrast, flours obtained from population rye cultivars exhibited lower FN values, ranging from 268 s for Dańkowskie Hadron and Piastowskie to 290 s for Dańkowskie Granat. These results are consistent with previous evaluations of the corresponding rye grain samples [15], which also showed that hybrid rye cultivars exhibited significantly higher FN values than population cultivars. Thus, the elevated FN values observed in hybrid grains were reflected in the corresponding flours, confirming a strong relationship between the enzymatic activity of the raw material and the technological properties of the milling product. The presented results indicate that FN, a key indicator of alpha-amylase activity, is strongly influenced by environmental factors such as harvest year and cultivation site, likely due to variations in climatic conditions affecting enzyme synthesis in the grain. Higher FN values observed in hybrid cultivars suggest lower enzymatic activity, which may enhance dough stability and improve baking performance in products where starch degradation needs to be limited. Future studies could investigate the relationship between FN values, dough rheology, and bread quality obtained from different rye cultivars, as well as explore strategies to optimize grain handling and processing to achieve desired FN levels.

Flour water absorption (WA) is a key technological parameter that determines the flour’s ability to bind and retain water. It plays a crucial role in baking technology, as it affects dough consistency, viscosity, gas-retention capacity, and the volume and structure of the bread crumb [62]. In rye flour, water absorption is primarily determined by the content of non-starch polysaccharides, especially arabinoxylans, which, owing to the presence of numerous hydrophilic groups, bind substantial amounts of water [63]. Increased WA in rye flour often correlates with higher protein content, which also binds water and thus contributes to its retention in the dough [64]. However, in rye flour, the role of protein in shaping WA is largely indirect. It depends mainly on the content and structure of arabinoxylans and on the activity of amylolytic enzymes, which modify the structure of starch and other components, thereby influencing the dough’s capacity to bind water [65,66]. Arabinoxylans, particularly the soluble fraction (WE-AX), exhibit high water-binding capacity and increase the viscosity of water extracts [67]. This results not only in higher WA but also in potential nutritional benefits. Higher WA associated with elevated levels of arabinoxylans and dietary fiber contributes to a greater proportion of fractions with potential prebiotic activity and may modulate the rate of starch digestion. Therefore, flours with higher WA may improve dough handling and gas retention while providing potential health benefits through increased dietary fiber and prebiotic fractions [68]. In the current study, the tested rye flours demonstrated WA values ranging from 59.0 to 68.6%. In comparison, Hansen et al. [34] found that wholemeal flour samples were characterized by higher WA values (63.8–70.6%). These differences may result from the use of flours with different extraction rates. In our study, the analyzed material consisted of flours with a 55% extraction rate, whereas Hansen et al. [34] examined wholegrain flours, which contain higher amounts of dietary fiber, including arabinoxylans that markedly influence the WA of rye flours [63,69]. Statistical results showed that all experimental factors significantly affected WA, with the most pronounced impact observed for the cultivation location of the rye grain used to produce the flours (Table 3). Flours made from rye grain from the Grabów site exhibited significantly higher WA (average 65.5%) compared with flours obtained from grain grown in Osiny, which showed WA values lower by 2.9 percentage points. Additionally, flour samples produced from rye grain harvested in 2020 had WA values that were 1.3 percentage points higher than those from grain harvested in 2019 (average 63.3%). Considering the cultivar factor, flours obtained from the hybrid rye Tur and the population Dańkowskie Turkus cultivars exhibited the lowest WA values (average 62.4% and 63.2%, respectively), whereas flour from the population cultivar Dańkowskie Skand showed the highest WA (average 65.5%).

### 3.4. Comprehensive Assessment of Flour Samples Using Principal Components Analysis (PCA)

**Figure 1:**
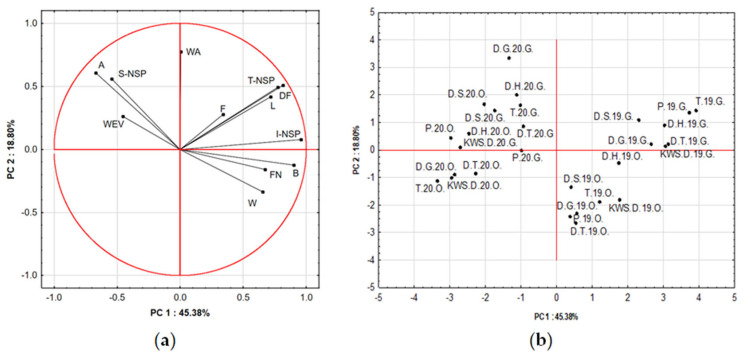
PCA results: (**a**) loading plot of variables for PC1 and PC2; (**b**) score plot representing the distribution of samples along the first two principal components. Explanations: sample codes in the chart (**b**) refer to rye grain from which were obtained rye flour: T (Tur), D.H. (Dańkowskie Hadron), D.S. (Dańkowskie Skand), D.G. (Dańkowskie Granat), D.T. (Dańkowskie Turkus), KWS.D. (KWS Dolaro), P (Piastowskie); numbers 19 and 20 refer to year of grain crop: 2019 and 2020, respectively; O and G refer to growing localization: Osiny and Grabów, respectively; codes of other indicators: WEV—water extract viscosity; WA—water absorption; A—ash content; T-NSP, S-NSP and I-NSP—total, soluble and insoluble non-starch polysaccharide content, respectively; F—fat content; DF—dietary fiber content; FN—falling number; B—protein content; W—moisture content; L—lignin content.

PCA was performed to evaluate the relationships among the analyzed quality parameters and to identify the main factors differentiating the samples. The first two principal components (PC1 and PC2) explained a total of 64.18% of the overall data variance, with PC1 accounting for 45.38% and PC2 for 18.80% (Figure 1). The loading plot (Figure 1a) revealed a clear grouping of variables into two main areas. PC1 was strongly and positively correlated with the total non-starch polysaccharides content (T-NSP), their insoluble fractions (I-NSP), dietary fiber content (DF), falling number (FN), and protein content (B) and negatively with ash content (A), water extract viscosity (WEV), and soluble non-starch polysaccharide (S-NSP) content. These variables contributed the most to the differentiation among samples. In contrast, flour water absorption (WA) and ash content were more strongly associated with PC2, indicating their smaller but still significant role in sample differentiation. A strong positive correlation was observed between T-NSP and S-NSP, as well as I-NSP and DF, confirming their shared contribution to the formation of the non-digestible carbohydrate fraction. The close alignment of the T-NSP, DF, and FN vectors indicates that samples with higher fiber content were also characterized by higher FN values, which may be associated with lower α-amylase activity. The ash content (A) and moisture content (W) parameters were positively related to the DF, indicating that samples with higher fiber content also contained more mineral substances and exhibited higher moisture levels. The score plot (Figure 1b) showed a clear separation of the analyzed samples in the factor space, confirming the effectiveness of PCA in distinguishing the studied groups. Samples from 2020 were mostly situated on the left side of the plot (negative PC1 values), while those from 2019 were positioned on the right (positive PC1 values). This separation confirms that the harvest year has a strong influence on measured quality traits, including fiber content, protein, and falling number.

## 4. Conclusions

The present study demonstrated that the harvest year exerted the greatest influence on the chemical composition and baking quality of the tested low-extraction rye flours (55% extraction rate). Flour samples obtained from grain harvested in 2019 exhibited significantly higher levels of protein, lipids, and dietary fiber. Flours from the 2020 harvest contained a higher proportion of soluble non-starch polysaccharides, resulting in increased water extract viscosity. The rye genotype also significantly affected both baking and chemical composition. Among the population cultivars, Dańkowskie Skand exhibited the most favorable baking characteristics, with the lowest falling number and the highest water absorption. In contrast, Dańkowskie Hadron exhibiting the highest levels of dietary fiber and its fraction (non-starch polysaccharides and lignin), as well as elevated water extract viscosity. The hybrid cultivars (Tur and KWS Dolaro) were associated with lower protein content and reduced amylolytic activity in comparison with the population cultivars. However, no significant differences were observed between hybrid and population cultivars in terms of dietary fiber content and its components. These findings suggest that both genotype selection and environmental factors are crucial determinants of the chemical and technological properties of rye flour. In particular, population cultivars, especially Dańkowskie Skand and Dańkowskie Hadron, may serve as valuable raw materials for producing organic rye flours. However, certain limitations of the present study should be acknowledged. The analyses were based on a limited number of technological and chemical indicators, which, although informative, do not fully capture the complexity of rye flour functionality. Moreover, the study focused on flours with a single extraction rate (55%), which may limit the applicability of the findings to flours produced at different milling fractions. Future research will include a broader set of analytical parameters, additional baking tests, and flours of varying extraction rates, providing a more comprehensive characterization of rye flour quality.

## Figures and Tables

**Table 1:** Major chemical constituents of the tested low-extract rye flour.

Parameter	Major Chemical Composition				
	Moisture Content (%)	Protein Content (% d.m.)	Ash Content (% d.m.)	Fat Content (% d.m.)	Carbohydrate Content (% d.m.)
Range	12.4–14.9	5.3–6.1	0.55–1.01	0.59–0.85	82.4–86.2
	Grain crop year				
2019	14.3 ± 0.6 ^b^	7.9 ± 0.3 ^a^	0.62 ± 0.05 ^b^	0.74 ± 0.06 ^a^	83.8 ± 0.1 ^b^
2020	13.5 ± 0.4 ^a^	6.2 ± 0.5 ^b^	0.85 ± 0.07 ^a^	0.71 ± 0.06 ^b^	84.7 ± 0.7 ^a^
	Grain growing location				
Osiny	14.0 ± 0.6 ^a^	6.8 ± 1.1 ^b^	0.70 ± 0.16 ^b^	0.71 ± 0.07 ^b^	84.9 ± 0.9 ^a^
Grabów	13.8 ± 0.7 ^b^	7.4 ± 0.8 ^a^	0.78 ± 0.15 ^a^	0.75 ± 0.05 ^a^	83.5 ± 0.6 ^b^
	Grain cultivar				
Tur	14.0 ± 1.0 ^b^	6.8 ± 0.3 ^d^	0.77 ± 0.16 ^a^	0.70 ± 0.06 ^d^	84.4 ± 1.1 ^b^
KWS Dolaro	13.8 ± 1.1 ^d^	6.6 ± 0.5 ^e^	0.76 ± 0.15 ^b^	0.65 ± 0.06 ^f^	84.9 ± 0.6 ^a^
Dańkowskie Granat	13.9 ± 0.9 ^c^	7.1 ± 0.3 ^c^	0.71 ± 0.12 ^f^	0.74 ± 0.06 ^c^	84.3 ± 1.2 ^bc^
Dańkowskie Hadron	13.8 ± 0.8 ^d^	7.3 ± 0.5 ^a^	0.75 ± 0.15 ^c^	0.78 ± 0.06 ^b^	83.7 ± 0.6 ^d^
Dańkowskie Skand	13.6 ± 1.0 ^e^	7.3 ± 0.3 ^a^	0.74 ± 0.12 ^d^	0.80 ± 0.06 ^a^	83.9 ± 1.1 ^cd^
Dańkowskie Turkus	14.4 ± 0.9 ^a^	7.2 ± 0.5 ^b^	0.71 ± 0.14 ^f^	0.68 ± 0.06 ^e^	84.2 ± 1.0 ^bc^
Piastowskie	13.8 ± 1.1 ^d^	7.3 ± 0.3 ^a^	0.73 ± 0.13 ^e^	0.77 ± 0.06 ^b^	84.1 ± 1.3 ^bc^
	ANOVA				
Factor	F_st._				
Crop year (A)	6024 **	16,615 **	35,614 **	149.1 **	349 **
Location (B)	81 **	1708 **	4872 **	140.8 **	837 **
Cultivar (C)	238 **	273 **	193 **	223.9 **	35 **
A × B	81 **	295 **	5 *	0.0 ^NS^	114 **
A × C	267 **	23 **	75 **	0.3 ^NS^	11 **
B × C	269 **	49 **	183 **	55.1 **	16 **
A × B × C	266 **	30 **	173 **	5.0 **	16 **

Note: The table shows data as ranges (minimum–maximum) and as mean values ± standard deviation; a–f—homogenous groups determined using t-Tukey’s test (*p* ≤ 0.05). Values marked with different letters differ significantly at *p* ≤ 0.05 (*), and *p* ≤ 0.01 (**); NS—not significant; F_st._—calculated value of the F-statistic. n = 3.

**Table 2:** Dietary fiber content and its components as well as water extract viscosity of the tested low-extract rye flour.

	Content (% d.m.)					WEV (mPa·s)
Parameter	DF	T-NSP	I-NSP	S-NSP	Lignin	
Range	6.24–8.63	5.57–7.51	2.10–4.22	2.89–4.40	0.45–1.12	3.37–6.35
	Grain crop year					
2019	7.52 ± 0.63 ^a^	6.66 ± 0.49 ^a^	3.53 ± 0.46 ^a^	3.13 ± 0.12 ^b^	0.87 ± 0.17 ^a^	4.40 ± 0.61 ^b^
2020	6.91 ± 0.44 ^b^	6.20 ± 0.41 ^b^	2.61 ± 0.25 ^b^	3.59 ± 0.36 ^a^	0.71 ± 0.12 ^b^	5.13 ± 0.67 ^a^
	Grain growing location					
Osiny	6.86 ± 0.39 ^b^	6.16 ± 0.34 ^b^	2.80 ± 0.46 ^b^	3.36 ± 0.29	0.70 ± 0.13 ^b^	4.74 ± 0.61 ^b^
Grabów	7.58 ± 0.60 ^a^	6.70 ± 0.50 ^a^	3.34 ± 0.60 ^a^	3.36 ± 0.41	0.88 ± 0.15 ^a^	4.80 ± 0.67 ^a^
	Grain cultivar					
Tur	7.39 ± 0.85 ^ab^	6.57 ± 0.65 ^ab^	3.09 ± 0.78 ^ab^	3.48 ± 0.23 ^ab^	0.82 ± 0.23 ^ab^	4.44 ± 0.46 ^d^
KWS Dolaro	7.03 ± 0.63 ^c^	6.28 ± 0.56 ^c^	3.12 ± 0.62 ^ab^	3.16 ± 0.22 ^c^	0.75 ± 0.09 ^c^	3.93 ± 0.42 ^e^
Dańkowskie Granat	7.20 ± 0.56 ^abc^	6.47 ± 0.39 ^abc^	2.92 ± 0.44 ^c^	3.55 ± 0.50 ^a^	0.72 ± 0.23 ^d^	4.96 ± 1.00 ^b^
Dańkowskie Hadron	7.47 ± 0.45 ^a^	6.63 ± 0.38 ^a^	3.18 ± 0.54 ^a^	3.45 ± 0.31 ^ab^	0.83 ± 0.15 ^a^	4.91 ± 0.11 ^b^
Dańkowskie Skand	7.15 ± 0.49 ^bc^	6.35 ± 0.38 ^bc^	2.96 ± 0.48 ^bc^	3.39 ± 0.34 ^ab^	0.80 ± 0.15 ^b^	5.21 ± 0.81 ^a^
Dańkowskie Turkus	7.23 ± 0.64 ^abc^	6.46 ± 0.49 ^abc^	3.15 ± 0.65 ^a^	3.31 ± 0.45 ^bc^	0.77 ± 0.15 ^c^	5.21 ± 0.31 ^a^
Piastowskie	7.08 ± 0.75 ^c^	6.24 ± 0.64 ^c^	3.06 ± 0.76 ^abc^	3.18 ± 0.27 ^c^	0.84 ± 0.15 ^a^	4.68 ± 0.79 ^c^
	ANOVA					
Factor		F_st._				
Crop year (A)	175.07 **	96.55 **	1262.22 **	261.81 **	1561.7 **	1292.1 **
Location (B)	243.99 **	139.48 **	434.55 **	0.02 ^NS^	1978.7 **	6.6 *
Cultivar (C)	6.64 **	5.72 **	7.94 **	15.42 **	70.0 **	290.8 **
A × B	38.41 **	17.79 **	48.81 **	0.22 ^NS^	531.3 **	704.3 **
A × C	10.58 **	12.81 **	15.30 **	10.04 **	84.8 **	114.1 **
B × C	11.43 **	6.45 **	18.16 **	9.98 **	247.1 **	39.6 **
A × B × C	7.61 **	5.17 **	5.76 **	14.71 **	48.9 **	68.2 **

Note: The table shows data as ranges (minimum-maximum) and as mean values ± standard deviation; a–e—homogenous groups determined using t-Tukey’s test (*p* ≤ 0.05). Values marked with different letters differ significantly at *p* ≤ 0.05 (*), and *p* ≤ 0.01 (**); NS—not significant; F_st._—calculated value of the F-statistic. n = 3. Abbreviations: DF—dietary fiber content; T-NSP, I-NSP and S-NSP—total, insoluble and soluble non-starch polysaccharide content, respectively; WEV—water extract viscosity.

**Table 3:** Technological quality of the tested low-extract rye flour.

Parameter	Falling Number (s)	Water Absorption (%)
Range	215–343	59.0–68.6
	Grain crop year	
2019	297 ± 20 ^a^	63.3 ± 2.1 ^b^
2020	264 ± 21 ^b^	64.6 ± 2.5 ^a^
	Grain growing location	
Osiny	271 ± 29 ^b^	62.4 ± 1.8 ^b^
Grabów	290 ± 20 ^a^	65.5 ± 1.8 ^a^
	Grain cultivar	
Tur	294 ± 25 ^a^	62.4 ± 2.4 ^d^
KWS Dolaro	294 ± 33 ^a^	64.6 ± 2.8 ^b^
Dańkowskie Granat	290 ± 21 ^b^	63.8 ± 3.5 ^c^
Dańkowskie Hadron	268 ± 26 ^d^	63.9 ± 1.8 ^c^
Dańkowskie Skand	271 ± 14 ^d^	65.5 ± 2.2 ^a^
Dańkowskie Turkus	280 ± 14 ^c^	63.2 ± 1.0 ^d^
Piastowskie	268 ± 35 ^d^	64.0 ± 1.8 ^c^
ANOVA		
Factor	F_st._	
Crop year (A)	1415.7 **	203 **
Location (B)	479.4 **	1508 **
Cultivar (C)	107.8 **	72 **
A × B	223.2 **	35 **
A × C	27.7 **	46 **
B × C	29.4 **	96 **
A × B × C	76.5 **	80 **

Note: The table shows data as ranges (minimum-maximum) and as mean values ± standard deviation; a–d—homogenous groups determined using t-Tukey’s test (*p* ≤ 0.05). Values marked with different letters differ significantly at *p* ≤ 0.01 (**); NS—not significant; F_st._—calculated value of the F-statistic. n = 3.

## Data Availability

The data presented in this study are available on request from the corresponding author (the data are not publicly available due to privacy or ethical restrictions).

## Supplementary Materials

The following supporting information can be downloaded at: https://www.mdpi.com/article/10.3390/foods15010003/s1, Table S1: Milling results of the tested rye grain.
