# Supplementary material for: The Chemical Composition and Baking Quality of Rye Flour from Grain with Organic Production
Source: Foods. 2025 Dec 19;15(1):3. doi: 10.3390/foods15010003 (PMC12786052; doi:10.3390/foods15010003)
Supplement: Supplementary file 1 [file foods-15-00003-s001.zip › foods-4018072-supplementary.pdf]

Table S1. Milling results of the tested rye grain.

	Fraction (%)				Composition of tested rye flour (flour yield of each sample 55%) (%)	
	Flour from reduction roll	Flour from breaking flour	Bran from reduction roll	Bran from breaking roll	Flour from reduction roll	Flour from breaking roll
**Grain harvest year**						
2019	52.1 ± 1.1	4.9 ± 0.7	16.0 ± 1.4	27.0 ± 1.3	52.1 ± 1.1	2.9 ± 1.1
2020	53.4 ± 1.4	5.2 ± 0.7	15.8 ± 1.0	25.6 ± 1.1	53.5 ± 1.3	1.5 ± 1.3
**Grain growing location**						
Osiny	52.1 ± 1.0	5.2 ± 0.7	16.4 ± 1.0	26.2 ± 0.9	52.1 ± 1.0	2.9 ± 1.0
Grabów	53.4 ± 1.4	4.9 ± 0.6	15.4 ± 1.2	26.3 ± 1.8	53.4 ± 1.5	1.6 ± 1.5
**Grain cultivar**						
Tur	53.0 ± 1.5	4.7 ± 0.2	16.3 ± 1.5	26.1 ± 1.1	53.0 ± 1.4	2.0 ± 1.5
KWS Dolaro	52.1 ± 1.2	4.9 ± 0.6	16.1 ± 1.1	26.9 ± 1.0	52.1 ± 1.0	2.9 ± 1.2
Dańkowskie Granat	53.2 ± 1.3	5.2 ± 0.8	14.4 ± 0.7	27.2 ± 1.5	53.2 ± 1.2	1.8 ± 1.3
Dańkowskie Hadron	53.1 ± 1.4	5.0 ± 0.6	15.9 ± 1.7	26.0 ± 2.7	53.1 ± 2.0	1.9 ± 1.4
Dańkowskie Skand	52.1 ± 1.7	5.4 ± 0.8	16.3 ± 0.7	26.2 ± 0.7	52.1 ± 1.2	2.9 ± 1.7
Dańkowskie Turkus	53.3 ± 1.1	5.1 ± 0.8	16.2 ± 1.0	25.4 ± 1.0	53.4 ± 0.7	1.6 ± 1.0
Piastowskie	52.5 ± 2.1	5.0 ± 1.0	16.4 ± 0.4	26.2 ± 1.3	52.5 ± 1.5	2.6 ± 2.1
